# Agreement of patient-measured intraocular pressure using rebound tonometry with Goldmann applanation tonometry (GAT) in glaucoma patients

**DOI:** 10.1038/srep42067

**Published:** 2017-02-06

**Authors:** Shaoying Tan, Marco Yu, Nafees Baig, Linda Hansapinyo, Clement C. Tham

**Affiliations:** 1 Department of Ophthalmology and Visual Sciences, The Chinese University of Hong Kong, Hong Kong; 2 Department of Ophthalmology, Chinese PLA General Hospital, Beijing, China; 3 Department of Mathematics and Statistics, Hang Seng Management College, Hong Kong; 4 Hong Kong Eye Hospital, Kowloon, Hong Kong; 5 Department of Ophthalmology, Faculty of Medicine, Chiang Mai University, Chiang Mai, Thailand; 6 Department of Ophthalmology and Visual Sciences, Prince of Wales Hospital, Shatin, Hong Kong

## Abstract

This study aims to determine the agreement of patient-measured intraocular pressure (IOP) using rebound tonometry with ophthalmologist-measured IOP using Goldmann applanation tonometry (GAT). Fifty-three glaucoma patients used rebound tonometry (Icare ONE, Icare Finland Oy., Finland) to measure their own IOP in ambient environments for 1 week, 5 times per day. Clinic IOP measurements were performed by ophthalmologists using GAT and by patients using rebound tonometry on examination days 1, 4 and 7 of the same week. The agreement between the two tonometries was evaluated by modified Bland-Altman plots and intra-class correlation coefficient (ICC) was determined. Differences in ICCs of them among the three examination days were evaluated by bootstrap resampling analysis. Respective within-measurement ICC of GAT and rebound tonometry were 0.98 and 0.94 on Day 1, 0.98 and 0.93 on Day 4, and 0.96 and 0.92 on Day 7. In a modified Bland-Altman plot, the mean difference ±1 standard deviation (SD) between the two tonometries was 0.15 ± 0.65 mmHg (*p* = 0.682). Between-measurement ICC were 0.66, 0.76 and 0.73 on the 3 examination days. There was no significant difference among ICCs. In conclusion, patient-measured IOP using rebound tonometry and ophthalmologist-measured IOP using GAT demonstrate good agreement.

Intraocular pressure (IOP) is a critical clinical parameter in the diagnosis and management of glaucoma. Goldmann applanation tonometry (GAT) has been considered as the gold standard for clinical IOP measurement due to its low intra- and inter-observer variability^1^. The principle of GAT is based on Imbert-Fick Law back to 1950^2^. Applanation refers to the central area of cornea that requires a force to flatten and balance the pressure inside the eye. However, it needs to be conducted by experienced ophthalmologists using a slit-lamp biomicroscope with good cooperation by the patients^3^. Besides, topical anesthesia and fluorescein staining are required in GAT, which further limit its use in non-clinic settings^3^,^4^. Since GAT is based on the principle of balancing the pressures on the cornea, the accuracy of GAT could also be affected by fluorescein, astigmatism, and corneal properties such as central corneal thickness (CCT)^5^,^6^. Development of new user-friendly devices is necessary to facilitate IOP measurement in the community, whether by eye care professionals, by carers, or by patients themselves.

Rebound tonometry is an IOP-measuring technology that is simple to use, and therefore could potentially be used by non-ophthalmologists and paramedical personnel^7^. Rebound tonometry contains a metal probe with a small plastic tip that can be bounced rapidly to the central cornea and rebound back. This procedure induces a current by the movements of probe in a magnetic coil. The rate of probe rebounding back induces a current that calculates the IOP^7^. Unlike GAT, rebound tonometry does not require the use of topical anesthesia and fluorescein staining during IOP measurement^8^,^9^,^10^. Although IOP measurement by rebound tonometry may also be influenced by CCT^11^,^12^,^13^ and other corneal properties^14^,^15^,^16^,^17^, it is reproducible and in good agreement with GAT^17^,^18^,^19^,^20^ as well as with other types of tonometries when performed by healthcare professionals^19^,^21^,^22^. A new generation of rebound tonometry, the Icare ONE (Icare Finland, Oy, Finland.), has been recently developed, which aims to allow measurement of IOP safely and accurately by glaucoma patients themselves outside the clinic^7^,^23^. Previous studies on rebound tonometry usually involved measurements conducted by ophthalmologists or eye care professionals. There were also some reports evaluating the correlation or agreement between rebound tonometry by patients or a third part, and GAT by healthcare professionals^24^,^25^,^26^,^27^,^28^, but all of the comparison were performed only once after the instruction of using the rebound tonometry, none of them investigated the repeatability of agreement over different days. Although Chen *et al*.^29^ evaluated the agreement between IOP measured by patients and GAT on two separate visited days, there was no comparison between agreements on the two study days, either the details on training of using the rebound tonometry. Moreover, the effectiveness of patient training and practice in rebound tonometry performed by patients themselves has not been evaluated in the published literature^7^,^26^,^30^,^31^,^32^,^33^.

In this study, we evaluated the consistency of patient-measured IOP using rebound tonometry over the course of one week after standardized training, and the results were compared to the GAT measurements by ophthalmologists at the beginning, middle and end of the study period.

## Patients and Methods

### Study subjects and ophthalmological examinations

Southern Chinese glaucoma patients with no previous ocular surgery (except laser peripheral iridotomy for primary angle closure glaucoma) were prospectively recruited at the eye clinics of the Chinese University of Hong Kong and Hong Kong Eye Hospital between April 2012 and December 2013. Informed consent was obtained from all study subjects. The study protocol was approved by the Ethics Committee for Human Research at the Chinese University of Hong Kong and in accordance with the tenets of the Declaration of Helsinki and the ICH-GCP guidelines.

All study subjects received at least primary school education, and they have normal mental and psychological abilities, with no difficulties in communications. They had either primary open angle glaucoma (POAG) or primary angle closure glaucoma (PACG). Angle status was determined by darkroom gonioscopy. The diagnosis of glaucoma was based on characteristic glaucomatous optic nerve head morphology, and confirmed by Humphrey automated perimetry (Humphrey Field Analyzer II, Carl Zeiss Meditec, California, USA; Central 24-2 threshold test, Swedish Interactive Thresholding Algorithm-standard strategy, size III white stimulus, with the foveal threshold test turned on). The criteria for glaucomatous visual field defect were adopted from a previous report^34^. All eyes with secondary causes of ocular hypertension or glaucoma, and eyes with previous ocular surgery (with the exception of laser peripheral iridotomy for PACG) were excluded.

One of the glaucomatous eyes from the patients was randomly selected using a random number table for inclusion in the study. If only one eye was diagnosed with glaucoma in a patient, it would be automatically included. The subjects received topical IOP-lowering eye drops, as clinically indicated.

Best-corrected Log MAR visual acuity and corneal pachymetry by ultrasonography were performed for all study eyes.

### Training program of IOP measurement by patients using rebound tonometry

IOP value, obtained from patient-conducted rebound tonometry using Icare ONE (Icare Finland, Oy, Finland), is the mean of six consecutive automatic measurements. A built-in inclination sensor detects probe errors or wrong positioning. Unreliable readings are automatically rejected. Indicator lights in 11 pressure zones between 5 and 50 mmHg could be displayed on the device, showing IOP values of 5–7, 7–10, 10–14, 14–18, 18–21, 21–24, 24–27, 27–30, 30–35, 35–40, and 40–50 mmHg. The time of measurement and the corresponding acceptable readings of IOP measurements were automatically stored in the device, and subsequently transferred to a computer through the proprietary Icare LINK software when the patient completed the study.

The training program on using rebound tonometry consisted of a 2-hour training session on the correct method for self-measurement of IOP, followed by a 7-day self-practice session.

All subjects were required to join the 2-hour training session individually for learning the usage of rebound tonometry. The training included watching a standard training video (http://www.youtube.com/watch?v=9Ov4VZXAZN4) with commentary by a study investigator who is experienced in using the self-rebound tonometry and who taught about practical tips. Subjects also had hands-on practices in using the tonometer under guidance and supervision. By the end of the training session, all subjects were assessed for their performance of the self-rebound tonometry to ensure that they followed the instructions in the standard operating procedure. A hard copy of Icare ONE Quick Guide was distributed to every patient after the training session.

Subjects would then move to the self-practice session and take the device back home to perform self-measurement of IOP using the rebound tonometry on the designated study eye for one week in a non-clinic setting, e.g. at home, office or school. A new, sterile single-use measurement probe was used for each measurement. Throughout the week, subjects were instructed to measure IOP at five different time points (i.e. at 08:00, 12:00, 16:00, 20:00, and 24:00) during the day. Each time, they were required to make 3 reliable measurements using the rebound tonometer and the time and data was automatically stored in the tonometer.

### Agreement evaluation of IOP measurement by patients using rebound tonometry and ophthalmologists using GAT in clinic

Clinic IOP was measured at the same time point on the first day, fourth day and the last day during the one-week study period. IOP readings were obtained by 2 different tonometries (GAT performed by an experienced ophthalmologist and rebound tonometry performed by patients themselves) in a randomized order within 30 minutes to avoid possible short-term IOP fluctuation. IOP measurement with GAT was performed at a slit-lamp biomicroscope on the selected eye after the application of one drop of 0.5% fluorescein sodium with 0.5% lidocaine. The reading in mm Hg was rounded to the next integer. Patients performed self-measurement of IOP using the rebound tonometer before or after GAT without supervision. All IOP measurements were performed in upright sitting position. Three measurements were taken from each instrument. The median of the three valid readings of each tonometry was used for statistical analysis of agreement evaluation. The patients and the ophthalmologists were not informed of the IOP results measured from the other tonometry.

### Feedbacks of patient training and self-measurement with rebound tonometry

A questionnaire was designed to obtain feedbacks from the study subjects on the clarity and adequacy of training, the general operability, as well as the perception of safety of self-measuring IOP with the rebound tonometry after the 1-week study period. A scoring system from 1 (very poor) to 5 (very good) was adopted. The questionnaire was conducted by the same study investigator for all patients. (Supplementary Document).

### Statistical analysis

Bland-Altman plots were used to assess the agreement between GAT and rebound tonometry for each evaluation session on the first day, fourth day and the last day during the one-week study period^35^, and the IOP measurement differences between GAT and rebound tonometry were evaluated by paired T-test. To adjust day effect in each individual, the modified Bland-Altman plot with repeated measures^36^ was used to assess the agreement between GAT and rebound tonometry by aggregating the clinical measurements obtained on the three examination days. The differences between GAT and rebound tonometry were evaluated by the analysis of variance (ANOVA) model:


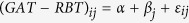


where (*GAT* − *RBT*)_*ij*_ represents the difference between GAT and rebound tonometry measured in day *i* for eye, *j* is the fixed effect representing the mean IOP difference between the 2 measurements, *β*_*j*_ is the random effect representing the eye-specific deviation of the IOP difference for eye *j*, and *ε*_*ij*_ represents the residual errors. The overall mean IOP difference was estimated by *α*. The 95% Limits of Agreement (LoA) were estimated by 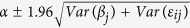. Agreement of within and between the two tonometries was determined by intra-class correlation coefficient (ICC). ICC >0.9 was defined as adequate, whereas ICC = 0.75–0.9 was defined as good, ICC = 0.5–0.75 as moderate, and ICC <0.5 as poor.

Resampling analysis was performed to assess the differences among agreements between the two tonometries on the three examination days, with 1,000 bootstrap replicates. A *p* < 0.05 was considered as statistically significant. All statistical analysis was performed using SPSS (version 20.0; SPSS Inc., Chicago, IL, USA) and R (version 2.15.2; R Foundation, Vienna, Austria).

## Results

### Demographic information of study subjects

Among fifty-three recruited glaucoma patients (31 PACG and 22 POAG; Table 1), 49 patients have completed the 1-week study, including the 3 clinic sessions. Three patients (1 PACG and 2 POAG) did not attend the Day 4 clinic session, and 1 patient (POAG) only attended the clinic on day 1. Among the 53 recruited patients, the percentage of completion of the required self-measurements was 86.0% ± 15.4% (mean ± standard deviation (SD); range, 37.1% to 100%; median, 88.6%). The demographic information of the study subjects was shown in Table 1.

### Agreement of IOP measurement by rebound tonometry with GAT

Mean IOP measurements by GAT ± 1 SD were 15.8 ± 4.5 mmHg, 15.5 ± 4.4 mmHg and 14.8 ± 4.1 mmHg on Day1, Day 4, and Day 7, respectively. Meanwhile, mean IOP measurements by rebound tonometry were 14.4 ± 4.9 mmHg, 15.8 ± 4.9 mmHg, and 15.5 ± 5.0 mmHg, on Day1, Day 4, and Day 7, respectively (Table 2).

Respective within measurement ICCs of GAT and rebound tonometry were 0.98 (95% confidence interval (CI): 0.97–0.99) and 0.94 (95% CI: 0.90–0.96) on Day 1, 0.98 (95% CI: 0.98–0.99) and 0.93 (95% CI: 0.89–0.96) on Day 4 and 0.96 (95% CI: 0.94–0.97) and 0.92 (95% CI: 0.87–0.95) on Day 7 respectively.

**Figure 1:**
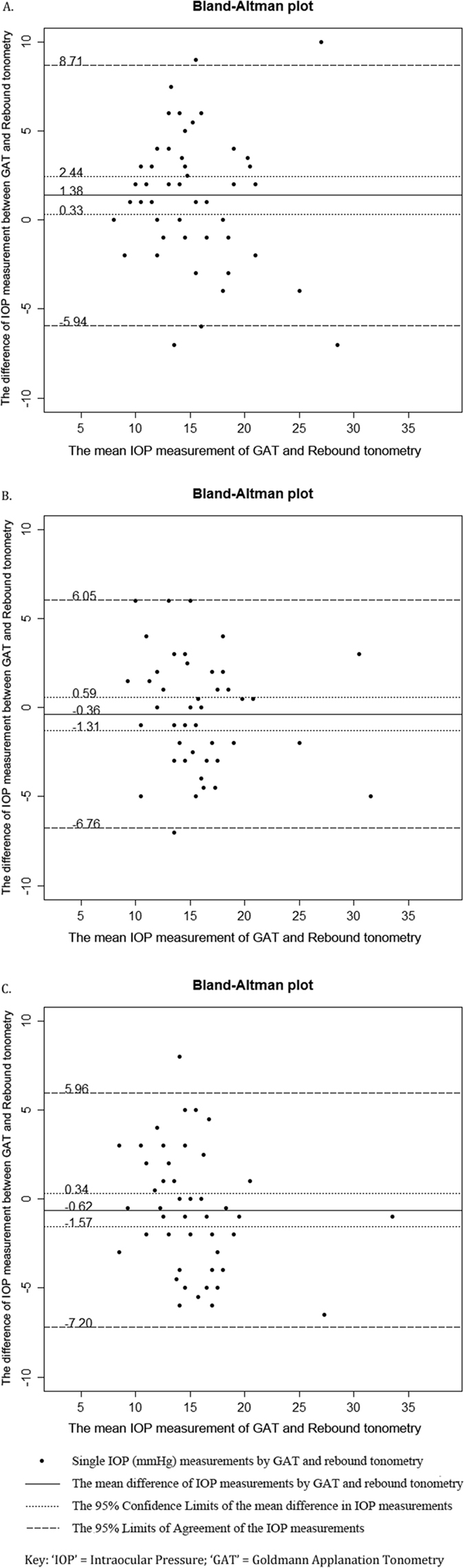
Bland-Altman plots of the mean difference of intraocular pressure measurement (mmHg) between Goldmann Applanation Tonometry and rebound tonometry. (**A**) Mean IOP differences on Day 1 were 1.38 mmHg; 95% confidence interval (CI): 0.33–2.44 mmHg; 95% limit of agreement (LoA): −5.94–8.71 mmHg (*p* = 0.010). (**B**) Mean IOP differences on Day 4 were −0.36 mmHg; 95% CI: −1.31–0.59 mmHg; 95% LoA: −6.76–6.05 mmHg (*p* = 0.448). (**C**) Mean IOP differences on Day 7 were −0.62 mmHg; 95% CI: −1.57–0.34 mmHg; 95% LoA: −7.20–5.96 mmHg (*p* = 0.195).

**Figure 2:**
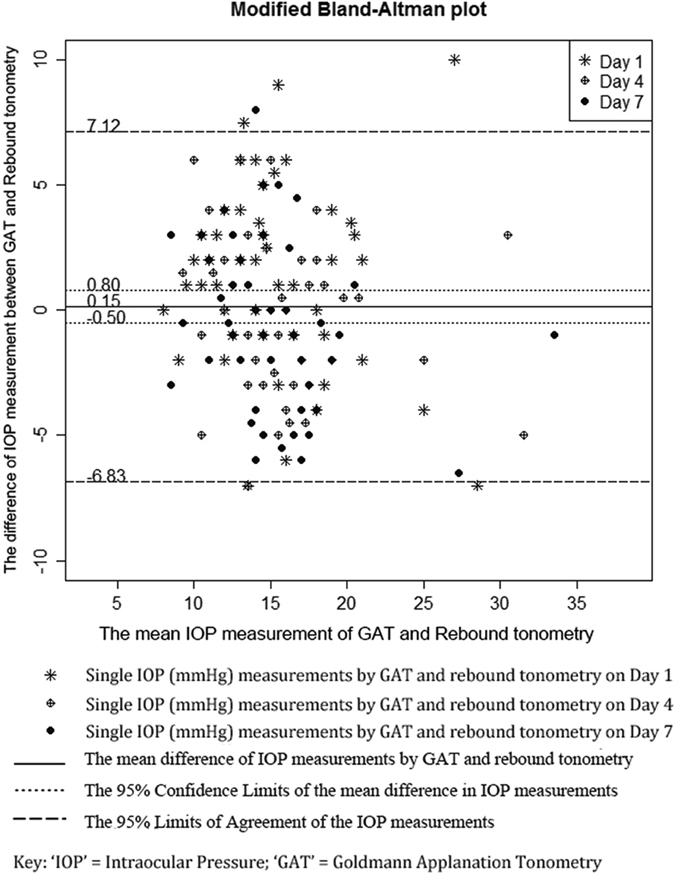
Modified Bland-Altman plots of the mean difference of intraocular pressure measurement (mmHg) between Goldmann Applanation Tonometry and rebound tonometry. Mean IOP differences on the three examination days were 0.15 mmHg; 95% confidence interval (CI): −0.50–0.80 mmHg; 95% limit of agreement (LoA): −6.83–7.12 mmHg (*p* = 0.682).

Bland-Altman plot showed that mean IOP differences were 1.38 mmHg on Day 1 (95% CI: 0.33–2.44 mmHg; 95% limit of agreement (LoA): −5.94–8.71 mmHg; paired t-test: *p* = 0.010; Fig. 1A), −0.36 mmHg on Day 4 (95% CI: −1.31–0.59 mmHg; 95% LoA: −6.76–6.05 mmHg; *p* = 0.448; Fig. 1B), and −0.62 mmHg on Day 7 (95% CI: −1.57–0.34 mmHg; 95% LoA: −7.20–5.96 mmHg; *p* = 0.195; Fig. 1C). By aggregating 3 days of measurements into a repeated measures ANOVA model, the mean IOP difference was 0.15 mmHg (95% CI: −0.50–0.80 mmHg; 95% LoA: −6.83–7.12 mmHg; *p* = 0.682; Fig. 2).

ICC between rebound tonometry and GAT measurements were 0.66 (95% CI: 0.46–0.79), 0.76 (95% CI: 0.61–0.86) and 0.73 (95% CI: 0.57–0.84) on the three examination days, respectively. Bootstrap resampling analysis further showed that there was no significant difference between the two tonometries on agreement on the 3 examination days (Table 3).

There were 45.5% (70 out of 154) of IOP measurements by GAT were higher than that by rebound tonometry. There was no significant difference in percentage of IOP measurements within 3 mmHg between GAT and rebound tonometry on the three examination days (62.2%, 71.4%, 67.3%, respectively; Fisher’s exact test: *p* = 0.574).

### Evaluation of training for self-measurement of IOP using rebound tonometry

Ninety-eight percent of the patients were satisfied with the instructions given in the training session, and 86% of patients were satisfied with the ease of operation of the rebound tonometry (Table 4). All patients were satisfied with the safety of the rebound tonometry, and no discomfort when using of the rebound tonometry was reported during the whole study period.

## Discussion

The Icare ONE tonometer, a new generation of rebound tonometry, is an electronic tonometer that enables IOP measurement by patients themselves in a non-clinic setting without anesthesia and fluorescein staining. In this study, all study participants could correctly use the device to obtain their own IOP after the standard training session, which is in agreement with the previous reports^26^,^27^,^28^,^30^,^37^.

Repeatability of GAT and rebound tonometry were analyzed by ICC in the current study. Respective within measurements ICC of GAT and rebound tonometry were 0.98 (95% CI: 0.97–0.99) and 0.94 (95% CI: 0.90–0.96) on Day 1, 0.98 (95% CI: 0.98–0.99) and 0.93 (95% CI: 0.89–0.96) on Day 4 and 0.96 (95% CI: 0.94–0.97) and 0.92 (95% CI: 0.87–0.95) on Day 7. These indicated that the repeatability of both GAT and rebound tonometry were good.

Previous studies demonstrated that IOP readings obtained by rebound tonometry were in good correlation and agreement with GAT^17^,^18^,^19^,^20^. However, the IOP measurements were taken by professional researchers using a rebound tonometer in those studies. A few studies documenting the agreement between patient-measured IOP using rebound tonometry (Icare ONE tonometer) with ophthalmologist-measured IOP using GAT in glaucoma patients^7^,^23^,^27^,^28^,^30^. One previous study showed the mean difference between IOP measured by patients using rebound tonometry (Icare ONE tonometer) and IOP measured by GAT was 0.8 mmHg, with 95% limits of agreement of −4.6 to 6.1 mmHg^23^. Another study showed significant difference between the two tonometries, with bias of 2.3 mmHg^30^. Although the recent study by Dabasia PL, *et al*.^28^ found that the mean difference between IOP measured by patients using rebound tonometry and GAT was 0.3 mmHg, with 95% limits of agreement of −4.6 to 5.2 mmHg, and they also presented the mean time required for training to obtain reliable self-measurement was 21 min (SD 5, range 11–30 min), the subsequent measurements were still under instruction. These studies have not discussed about the importance of effective training and practice for a longer-term (1-week) non-clinic self-measurement in the use of rebound tonometry by patients.

In this study, the mean IOP difference between rebound tonometry performed by patients and GAT performed by ophthalmologist were aggregated, and adjusted by 3 different measurement days, was 0.15 ± 0.65 mmHg (95% LoA: −6.83–7.12 mmHg; Fig. 2). ICC analyses between rebound tonometry and GAT readings were 0.66 (95% CI: 0.46–0.79), 0.76 (95% CI: 0.61–0.86) and 0.73 (95% CI: 0.57–0.84) on the 3 examination days, respectively. The agreement between GAT and rebound tonometry in our study was also comparable to previous studies in which both tonometries were conducted by professional examiners^3^,^19^,^38^,^39^,^40^. Moreover, previous studies suggested that the measurement techniques could influence the rebound tonometry readings^9^,^15^, implying that different readings were obtained from patients and experienced professionals^9^,^22^,^30^. In our study, larger variation of agreement was not observed when compared to other studies. In addition, bootstrap resampling analysis further showed that there was no significant difference between the two tonometries on agreement for the three examination days (Table 3). These findings indicated that the quality of IOP measurement by rebound tonometry was not affected by the environment or experiences of the users, and appeared to be quite stable during the whole week of study.

Furthermore, the differences of within 3 mmHg between self-IOP measurement by rebound tonometry and clinic IOP measurement by GAT in glaucoma patients were 62.2%, 71.4% and 67.3% on the three examination days, respectively. This is comparable to other studies (62.7–80%)^1^,^7^,^41^. This suggested that rebound tonometry is clinically acceptable, especially when taking into consideration of the greater-than-2 mmHg calibration error in GAT^1^,^3^,^7^,^41^.

Although there are studies showing that IOP values may decrease with closely repeated measurements, possibly due to the effect of repeated corneal contact and pressure^7^, we did not observe this phenomenon in our study. To avoid the effect of repeated measurements on IOP, we randomized the measurement order of GAT and rebound tonometry in clinic. Several studies suggested that IOP readings from rebound tonometry could be affected by CCT^11^,^12^,^13^ and other corneal biomechanical properties^14^,^15^,^16^,^17^. Studies by Avitabile *et al*.^42^ and Marini *et al*.^43^ on normal, ocular hypertension and glaucoma subjects, and study by Dahlmann-Noor *et al*.^44^ involving 102 children all showed that rebound tonometry gave significantly higher IOP readings than GAT and that the disagreement between rebound tonometry and GAT increased with an increase in CCT. In this study, however, the correlation with CCT and the clinical parameters may not be detected with the limited sample size. Furthermore, IOP measured by rebound tonometry of older generations was reported to be overestimated by 1.34 to 1.8 mmHg, when compared to GAT^38^,^39^,^40^. Although Pearson correlation coefficients were high (>0.8) and mean differences between rebound tonometry and GAT were small (0.79 to 1.5 mmHg), the correlation was not as good in eyes with higher IOP (23 to 60 mmHg). In our study, the subjects had IOP medically controlled to within a tight range (15.2 ± 4.43 mmHg by GAT). The correlation in subjects with high IOP could not be addressed in this study. Further studies are necessary to determine the influences of corneal biomechanical properties on IOP measurement, as well as the correlation with GAT and rebound tonometry in patients with high IOP.

The IOPs were significantly different between rebound tonometry and GAT on day 1 (paired t-test: *p* = 0.010), but not on day 4 (*p* = 0.448) and day 7 (*p* = 0.195). ICC between rebound tonometry and GAT measurements were 0.66 (95% CI: 0.46–0.79), 0.76 (95% CI: 0.61–0.86) and 0.73 (95% CI: 0.57–0.84) on the three examination days, respectively, this implies that the agreements might be better on day 1. However, bootstrap resampling analysis further showed that there was no significant difference between the two tonometries on agreement on the 3 examination days. The familiarity might increase the accuracy of rebound tonometry, and a 2-hour training session might be good enough for patients to handle the rebound tonometry by themselves.

Importantly, IOP measurement by patients using rebound tonometry was well accepted by over 80% of the participants in our study, without any discomfort or adverse event. Majority of the patients were satisfied with the instructions, ease of usage, and the safety of rebound tonometry, indicating that rebound tonometry may be used by patients themselves for self-measurement of IOP outside the clinic setting. Furthermore, identification of IOP fluctuations by rebound tonometry in non-clinical environment may be warranted, since IOP fluctuation may be an independent risk factor for glaucoma progression^45^,^46^,^47^. A pattern was observed in the diurnal IOP profiles: the mean patient-measured IOP was highest in the morning, gradually decreased over the course of a day, and was lowest by midnight (*p* < 0.001). No statistically significant differences in IOP values across the days were found.

The findings from this study confirm good agreement between patient-measured IOP using rebound tonometry and ophthalmologist-measured IOP using GAT. With this knowledge, patient-measured IOP using rebound tonometry may provide clinicians additional IOP control data for clinical decision making, in both patients with ‘chronic’ glaucoma and ocular hypertension. In suspected cases of normal tension glaucoma, patient-measured IOP using rebound tonometry may also help confirm whether episodes of ocular hypertension outside the clinic could have been missed. In patients with episodic acute ocular hypertension, e.g. patients with Possner Schlossman Syndrome or uveitic glaucoma, patients may be trained to conduct self-IOP measurements with rebound tonometry, and to seek immediate or early ophthalmic attention should their IOP rises. Patient-measured IOP may also have implications on treatment strategies in many different ways. For example, if significant IOP fluctuations and high peak IOPs are identified by patient-measured IOP, treatment measures with higher efficacy in flattening the IOP profile, such as trabeculectomy, may be considered.

Our study had the following limitations. Firstly, we adopted the IOP measurement by GAT as a ‘gold standard’. Even though GAT may be the tonometric method most widely adopted in clinical practice by ophthalmologists, it may not be the most accurate method to measure IOP^1^,^3^,^48^. Therefore, in this study, we aimed at assessing the agreement between rebound tonometry and GAT, instead of how rebound tonometry performed to determine true IOP. Secondly, both GAT and rebound tonometry required some degree of cooperation by the study subjects. Differences in mental status, manual dexterity and education level, could lead to bias, but these were not quantified. In our study, we aimed to minimize this bias with a standard and adequate training program for all patients. The IOP measurements by rebound tonometry during the whole study period were shown to be consistent. Moreover, the doctor who performed GAT was masked from the rebound tonometry measurement to avoid observer bias. Thirdly, we could ensure strict adherence to the measurement protocol when the patients performed self-IOP measurement in our clinic, but we could not ensure such adherence once the patients had left our facilities. Since 6 readings were collected for each IOP measurement, rebound tonometry is able to recognize wrong position or large variation and automatically delete possibly unreliable readings. Only the more consistent readings would be stored, allowing us to ensure some degree of compliance with the measurement protocol. Correct usage of the device was also re-assessed in our clinic on Day 4 and Day 7 of the study week. Fourthly, IOP measurements by rebound tonometry were only performed by patients in this study because of the difficulties to differentiate between IOP measurements performed by the patient from a clinician when downloading the readings from the device. It will lead to a limitation on investigating the agreement between ophthalmologist and patient performed rebound tonometry. The subjects recruited in this study were all relatively young and under treatment with controlled IOP. Conclusions from this study could therefore not be extrapolated to dissimilar situations, such as to subjects with very old or young age, very thick or thin CCT, with very high or low IOP, or doing different activities. The daily activities of retirees were mostly with the home, while the younger recruits were actively working, with occupations including teachers, nurses, civil servants, etc. They can take the measurements in their place of work. People engaging regularly in outdoor work or activities were not recruited, because it would be difficult for them to follow the study instructions Further studies are warranted to evaluate the performance of rebound tonometry, relative to GAT, under such special situations. Moreover, healthy control group was not included in this study. Further study was needed to compare IOP measurement by rebound tonometry between glaucoma patients and normal health controls as well.

In summary, this study determined the repeatability and consistency of IOP measurement by glaucoma patients themselves using rebound tonometry, which demonstrated good agreement with the IOP measurement by GAT performed by eye care professionals. After a 2-hour training session and 1-week practice, patients could handle the rebound tonometer well by themselves. This device may be suitable for self-measurement of IOP by glaucoma patients outside the clinic setting.

## Additional Information

**How to cite this article**: Tan, S. *et al*. Agreement of patient-measured intraocular pressure using rebound tonometry with Goldmann applanation tonometry (GAT) in glaucoma patients. *Sci. Rep.* **7**, 42067; doi: 10.1038/srep42067 (2017).

**Publisher's note:** Springer Nature remains neutral with regard to jurisdictional claims in published maps and institutional affiliations.

## Supplementary Material

Supplementary Information

## Figures and Tables

**Table 1:** Demographic information of the study population.

	n	n
Number of subjects	POAG: 22	PACG: 31
Gender	Male: 28	Female: 25
Studied eye	Right: 27	Left: 26
Cataract	Yes: 30	No: 23
Previous AAC	Yes: 11	No: 42
Previous PI.	Yes: 30	No: 23
	Mean ± SD	Range
Age (year)	59.5 ± 11.6	34.1–85.6
Number of drugs (n)	2.4 ± 1.5	0–5
Spherical equivalent (Diopter)	−0.96 ± 4.49	−10.50–5.00
BCVA (LogMAR)	0.8 ± 0.4	0.1–2.0
CCT (μm)	541.8 ± 33.3	473.0–632.0
C/D Ratio	0.7 ± 0.2	0.3–1.0

Key: n: number of subjects.

SD: Standard Deviation.

POAG: Primary Open Angle Glaucoma.

PACG: Primary Angle Closure Glaucoma.

AAC: Acute Angle Closure.

PI: Peripheral Iridotomy.

BCVA: Best-Corrected Visual Acuity, LogMAR.

CCT: Central Corneal Thickness.

C/D Ratio: Vertical Cup-to-Disc Ratio.

**Table 2:** Intraocular pressure measurements by Goldmann applanation tonometry and rebound tonometry on three examination days.

	Mean	SD	Minimum	Median	Maximum
**GAT**					
Day1	15.8	4.5	8.0	16.0	32.0
Day4	15.5	4.4	8.0	15.0	32.0
Day7	14.8	4.1	7.0	14.0	33.0
**Rebound tonometry**					
Day1	14.4	4.9	8.0	13.0	32.0
Day4	15.8	4.9	7.0	16.0	34.0
Day7	15.5	5.0	7.0	15.0	34.0

Key: GAT: Goldmann Applanation Tonometry; SD: Standard Deviation.

**Table 3:** Bootstrap resampling analysis for difference of intraclass correlation coefficients between Goldmann Applanation Tonometry and rebound tonometry on three examination days.

	Difference (ICC)	*p*-value	95% CI
Day1–Day4	−0.08	0.458	−0.22–0.18
Day1–Day7	−0.07	0.594	−0.25–0.19
Day4–Day7	0.00	0.964	−0.16–0.16

Key: CI: Confident Intervals; ICC: Intraclass Correlation Coefficient.

**Table 4:** Questionnaire evaluation of self-IOP measurement by rebound tonometry and the training program provided.

	Degree	Instruction clarity	Instruction adequacy	Ease of Use	Safety of use
Very Poor	1	2%	2%	2%	0%
	2	0%	0%	12%	0%
Fair	3	32%	34%	44%	17%
	4	56%	56%	35%	71%
Very Good	5	10%	8%	8%	12%

Remark: no subject experienced any uncomfortable feelings or injury.
