# Supplementary material for: Agreement of patient-measured intraocular pressure using rebound tonometry with Goldmann applanation tonometry (GAT) in glaucoma patients
Source: Sci Rep. 2017 Feb 6;7:42067. doi: 10.1038/srep42067 (PMC5292696; doi:10.1038/srep42067)
Supplement: Supplementary Information [file srep42067-s1.pdf]

Agreement of patient-measured intraocular pressure using rebound tonometry with Goldmann applanation tonometry (GAT) in glaucoma patients

[1,6]Shaoying TAN, Ph.D., MD,

[1,4]Marco YU, PhD,

[1,2]Nafees BAIG, MRCS, FCOphth(HK),

[1,2,5]Linda HANSAPINYO, MD,

[1,2,3]Clement C THAM, FRCS, FCOphth(HK)

[1]Department of Ophthalmology and Visual Sciences, The Chinese University of Hong Kong, Hong Kong;

[2]Hong Kong Eye Hospital, Kowloon, Hong Kong;

[3]Department of Ophthalmology and Visual Sciences, Prince of Wales Hospital, Shatin, Hong Kong.

[4]Department of Mathematics and Statistics, Hang Seng Management College, Hong Kong;

[5]Department of Ophthalmology, Faculty of Medicine, Chiang Mai University, Chiang Mai, Thailand;

[6]Department of Ophthalmology, Chinese PLA General Hospital, Beijing, China.

Patient Questionnaire on safety and ease of use of ICare rebound self-tonometry

Do you find instructions of the manual clear?

☐ Very unclear ☐ Unclear ☐ Fair ☐ Clear ☐ Very Clear

Do you find instructions of the manual adequate?

☐ Very Inadequate ☐ Inadequate ☐ Fair ☐ Adequate ☐ Very Adequate

Do you find the ICare rebound self-tonometry easy to use?

☐ Very Difficult ☐ Difficult ☐ Fair ☐ Easy ☐ Very Easy

Do you find the ICare rebound self-tonometry safe to use?

☐ Very Unsafe ☐ Unsafe ☐ Fair ☐ Safe ☐ Very Safe

Have you ever had any eye injury while using ICare rebound self-tonometry?

☐ Yes ☐ No

If yes, please write down the detail of injury.

____
